# Assessment of the biopotency of follitropin alfa and lutropin alfa combined in one injection: a comparative trial in Sprague-Dawley rats

**DOI:** 10.1186/1477-7827-6-31

**Published:** 2008-07-22

**Authors:** Michael Alper, Randal Meyer, Chris Dekkers, Diego Ezcurra, Joan Schertz, Eduardo Kelly

**Affiliations:** 1 Boston IVF, Waltham, MA, USA; 2 Sioux Biochemical, Inc., Sioux Center, IA, USA; 3 EMD Serono, Inc. (an affiliate of Merck KGaA, Darmstadt, Germany), Rockland, MA, USA

## Abstract

**Background:**

The current study was designed to determine if follitropin alfa (recombinant human follicle-stimulating hormone; r-hFSH) and lutropin alfa (recombinant human luteinizing hormone; r-hLH) biopotencies were unchanged by reconstituting in sterile water for injection and mixing prior to injection.

**Methods:**

The biopotencies of r-hFSH and r-hLH were determined following injection of female Sprague-Dawley rats with a mixture of follitropin alfa revised formulation female (RFF) and lutropin alfa (1:1, r-hFSH:r-hLH). Biopotencies of follitropin alfa RFF and lutropin alfa were measured using ovarian weight and ascorbic acid depletion assays, respectively, and compared with a reference standard. Stock mixtures of follitropin alfa RFF and lutropin alfa (1:1) were prepared within 1 h prior to each respective assay's injection and stored at 6 +/- 2°C. Separate low dose (follitropin alfa RFF 1.5 IU/rat, lutropin alfa 2 IU/rat) and high dose (follitropin alfa RFF 3 IU/rat, lutropin alfa 8 IU/rat) treatments were prepared from stock mixtures or individual solutions by diluting with 0.22% bovine serum albumin saline solution and injected within 1 h of preparation. The main outcome measures were ovarian weight and ovarian ascorbic acid depletion.

**Results:**

FSH bioactivities were similar (p > 0.10) between the individual follitropin alfa RFF test solution (84.2 IU) and follitropin alfa RFF/lutropin alfa (87.6 IU) mixtures prepared within 1 h of injection and stored at 6 +/- 2°C. LH bioactivities were similar (p > 0.10) between lutropin alfa (94.7 IU) test solution and lutropin alfa/follitropin alfa RFF (85.3 IU) mixtures prepared within 1 h of injection and stored at 6 +/- 2°C for not more than 1 h prior to injection.

**Conclusion:**

Mixing follitropin alfa RFF and lutropin alfa did not alter the bioactivity of either FSH or LH.

## Background

In 1988, human follicle-stimulating hormone (hFSH) was successfully expressed using Chinese hamster ovary cells, representing the initial steps for commercial development of gonadotropin products originating from recombinant deoxyribonucleic acid (r-DNA) technology [1]. The first recombinant hFSH, GONAL-f^® ^(r-hFSH, follitropin alfa for injection), was licensed in 1995 for marketing in the European Union [2]. By the turn of the century, recombinant human luteinizing hormone (r-hLH, lutropin alfa for injection, Luveris^®^) and recombinant human chorionic gonadotropin (r-hCG, choriogonadotropin alfa injection, Ovidrel^®^) were also commercially produced, marking the availability of all three human recombinant gonadotropins for infertility treatment.

Until the successful introduction of the recombinant proteins, gonadotropin products for the treatment of infertility consisted of a partially purified mixture of urine-derived FSH, LH and/or chorionic gonadotropin (hCG). While the urine-derived products have long represented the mainstay of infertility therapy, the recombinant products alone provide the distinct advantage of solo administration. That is, unlike the urine-based derivatives, r-hFSH and r-hLH can be administered either independently or in combination, in ratios that are static or vary during the course of therapy. For the clinician, dosing flexibility of the individual gonadotropins offers the potential to tailor treatment according to the patient's distinct gonadotropin requirements. The use of individualized protocols to stimulate follicular development reflects concepts originally elaborated by Brown (the FSH threshold) [3] and Hillier (the LH ceiling) [4], and builds on scientific knowledge regarding the continuum of follicular development through an FSH-dependent phase and a period of LH-responsive maturation [4,5]. In fact, numerous recent clinical investigations have focused on controlled ovarian stimulation (COS) protocols that incorporate LH activity at various doses and phases in the treatment cycle [5-8].

The potential disadvantage of utilizing a combinative approach, however, is that the recombinant gonadotropin products are generally administered as separate injections. The inconvenience of preparing for and administering two injections may be considerable, depending on the prescribed daily dose. Some healthcare providers have instructed their patients on methods for mixing the products together, thereby obviating the need for a second injection [9]. Clinical outcomes appeared to be unaffected by this practice.

le Cotonnec and colleagues compared the potential interactions of r-hLH with r-hFSH when administered individually or combined in one injection in a prospective, randomized, Phase I, crossover study of 12 healthy, pituitary down-regulated women [10]. Patients received single doses of 150 IU r-hFSH plus 150 IU r-hLH, alone or combined in a single syringe, followed by 150 IU r-hFSH plus 150 IU r-hLH, alone or combined in a single syringe once daily for 7 days. No pharmacokinetic (PK) interactions between the products were observed after single dosing or in combination. Furthermore, no correlations were found between maximum serum FSH or LH concentrations and the pharmacodynamic (PD) responses recorded, including serum estradiol and inhibin concentrations, and ovarian follicular growth. The authors concluded that there were no PK or PD differences between separate and combined administration of r-hLH and r-hFSH.

In the current study, the objective was to determine if lower doses (75 IU r-hFSH and 75 IU r-hLH) of individual follitropin alfa revised formulation female (RFF) and lutropin alfa biopotencies remained unchanged after mixing the products together, assessing the outcomes via *in vivo *bioassays.

## Methods

### Study design

Follitropin alfa RFF biopotency was determined using the traditional Steelman-Pohley hCG augmentation assay, which measures ovarian hypertrophy resulting from exogenous FSH treatments of immature female rats when administered in conjunction with hCG [11]. According to regulatory requirements, FSH biological activity of FSH-containing gonadotropins is assessed by the same Steelman-Pohley bioassay used in this study [12-15]. Biopotency of lutropin alfa was determined using the ovarian ascorbic acid depletion assay, which measures the decrease in ovarian ascorbic acid in response to exogenous LH treatments administered to pseudo-pregnant rats [16]. This assay was chosen due to its greater sensitivity for detecting LH compared to the hyperemia, ventral prostate, and interstitial cell assays. Moreover, the ovarian ascorbic acid depletion assay is 8-, 33-, and 40-fold more sensitive, respectively, than the aforementioned assays [16]. Although the Van Hell seminal vesicle weight gain bioassay is designated by regulatory authorities to determine the LH biological activity of LH-containing gonadotropin products, the ovarian ascorbic acid depletion assay was used in this analysis for the reasons described [16,17].

### Reference standards

The National Institute of Biological Standards and Controls (NIBSC) Urinary Follicle Stimulating Hormone and Urinary Luteinizing Hormone, 4^th ^International Standard, NIBSC Code: 98/704 (NIBSC, Herts, UK) reference standard was prepared for each replicate assay by dissolving a single vial (FSH assay) or two vials (LH assay) each with 1.0 ml sterile saline solution and subsequently pooled, in the case of the latter. By definition of the World Health Organization Expert Committee on Biological Standardization, the NIBSC 98/704 standard contains 72 IU of urinary FSH and 70 IU of urinary LH per ampoule [18]. The NIBSC 98/704 standard was used because a similar standard was not available for a combination of recombinant FSH and recombinant LH.

### Test animals

Twenty-two-day-old female Sprague-Dawley rats (Harlan, Indianapolis, IN) were used for each bioassay (FSH assay, n = 121; LH assay, n = 112). Each treatment group was housed in individual cages, 30 × 40 cm, in a temperature-controlled room (22 ± 2°C). Animals were given access to water *ad libitum*, and fed a rat pellet consisting of 19% crude protein, ≥ 5.0% crude fat, ≤ 5.0% crude fiber. The supplier certified the animals to be free of common viruses, bacteria, and parasites. All animal studies were approved by the Institutional Animal Care and Use Committee.

Commercial products were supplied by EMD Serono, Inc. (Rockland, MA, USA). Each 75 IU follitropin alfa RFF for injection (GONAL-f^® ^RFF) and 75 IU lutropin alfa for injection (Luveris^®^) mono-dose vial was overfilled to accommodate potential reconstitution and administration losses to insure delivery of the 75 IU dose [12,17]. As determined by the respective Steelman-Pohley and Van Hell bioassays, the release specifications for the commercial product used was FSH = 75.5 IU/vial (GONAL-f^® ^RFF batch number 1970606D01) and LH = 94.2 IU/vial (Luveris^® ^batch number 5150911D) (data on file with Merck Serono International S.A., Geneva, Switzerland). Three 75 IU mono-dose vials of follitropin alfa RFF and five 75 IU mono-dose vials of lutropin alfa were reconstituted with their accompanying diluent (1 ml Sterile Water for Injection [SWFI]) for each assay. The reconstituted products were pooled to provide sufficient stock material for each replicate assay, e.g. 3 ml for follitropin alfa RFF and 5 ml for lutropin alfa. Stock mixtures of follitropin alfa RFF and lutropin alfa (1:1) were prepared within 1 h prior to each respective assay's injection and stored at 6± 2°C. The 1:1 ratio of follitropin alfa RFF and lutropin alfa used in the assay is consistent with the NIBSC 98/704 standard of urinary FSH and urinary LH used in this assay.

Preliminary FSH and LH assays were conducted to determine the optimum linear response dosages for the respective assays. Separate low dose (follitropin alfa RFF 1.5 IU/rat, lutropin alfa 2 IU/rat) and high dose (follitropin alfa RFF 3 IU/rat, lutropin alfa 8 IU/rat) treatments were prepared from stock mixtures or individual solutions by diluting with 0.22% bovine serum albumin saline solution and injected within 1 h of preparation. The FSH assay was conducted in duplicate, utilizing 8 rats per dose per replicate assay, totaling 16 rats per dose per assay. The LH assay was conducted in triplicate, utilizing 5 rats per dose per replicate assay, totaling 15 rats per dose per assay.

### FSH bioassay

For the FSH bioassay, 121 23-day-old Sprague-Dawley rats were injected with test solution, twice daily, for 3 days. Standard doses of test solutions (regardless of body weight) were used for practicality. Respective high and low doses were diluted in diluent containing hCG and administered subcutaneously using a 1 cc syringe equipped with a 1/2 inch × 25 1/2 gauge needle (Monoject, Sherwood Medical, St. Louis, MO) in 0.5 ml injection volumes at 8 am and 5 pm. The total hCG dose was 20.5 IU/rat. Animals were euthanized 74 ± 2 h after the initial injection, ovaries were removed and excised free of fat and connective tissue, and promptly weighed using a Sartorius scale (model BP 61, Sartorius Corp., Edgewood, NY) to the nearest 0.1 mg. Test solutions were quantified against the reference standard.

FSH bioactivity was calculated according to the United States Pharmacopeia (USP), utilizing ovarian weights according to the following formula:

M' = cih'T_a_/2ΣT_b_

where: M' = the log-relative potency of each unknown;

c = the constant taken from Table 6 in USP 28-NF [19];

i = the interval in logarithms between successive log-doses;

h' = the number of values of T_b _summed in the denominator;

T_a _= T_i _for the difference in the responses to the standard and to the unknown;

T_b _= T_i _for the combined slope of the dosage response curves for the standard and unknown [19].

Confidence intervals (CI) were calculated according to USP 28-NF [19].

### LH bioassay

For the LH bioassay, 112 26-day-old Sprague-Dawley rats were pre-treated with 50 IU/rat pregnant mare serum gonadotropin (PMSG). At 29 days of age, 62 ± 2 h after PMSG treatment, hCG, 25 IU/rat, was injected to induce pseudo-pregnancy. Nine days following hCG administration (aged 39 days), rats were weighed and treated with test solutions intravenously (using 2.8 IU per 100 g body weight). Rats were lightly anesthetized with ether, and drug was injected into the tail vein using a 1 cc syringe (Monoject, Sherwood Medical) equipped with a 1/2 inch × 25 1/2 gauge needle. The 2 and 8 IU doses were administered in a 0.5 ml solution per 100 g body weight.

Following a 4 h ± 10 min incubation period, rats were euthanized, ovaries removed and promptly excised free of fat and connective tissue, blotted on filter paper, and weighed to the nearest 0.1 mg. Mean (SD) rat weight was 124.3 (7.1) g. Ovaries were immediately homogenized in 10.0 ml 2.5% metaphosphoric acid. Tissue homogenate was assayed for ascorbic acid concentration using the Parlow modification of Mindlin and Butler [20].

Test solutions were quantified against the reference standard. USP LH bioactivity was calculated using ascorbic acid concentrations according to the same formula used for the FSH bioactivity.

### Statistical analysis

According to the USP, FSH or LH potency is adequate if it falls within a range of 80–125% of the labeled potency and if the confidence interval is not >1.8 [19]. When the confidence limit from replicate assays was determined, it was <0.18, and therefore no data was omitted from analysis because of exceeding the CIs. Data were analyzed using ANOVA procedures to examine differences among reference standard, individual gonadotropins, and the follitropin alfa RFF/lutropin alfa mixture treatment groups. Since no significant differences were found, treatment means were not separated.

## Results

FSH bioactivities (Table 1) of the individual follitropin alfa RFF test solution and follitropin alfa RFF/lutropin alfa (1:1) mixtures stored at 6 ± 2°C within 1 h prior to injection were similar (p > 0.10). The mean FSH IU for the FSH standard was 72 IU/vial (range, 65.4–79.3).

**Table 1:** Follicle-stimulating hormone (FSH) bioactivities (mean and 95% confidence interval)

	Follitropin alfa RFF (n = 37)	Follitropin alfa RFF/lutropin alfa mixture (1:1) (n = 32)	
FSH bioactivity per vial (IU of NIBSC 98/704)	84.2 (75.5–92.7)	87.6 (79.1–96.5)	p > 0.10

RFF, revised formulation female.

LH bioactivities (Table 2) were not statistically different between the individual lutropin alfa test solution and lutropin alfa/follitropin alfa RFF (1:1) mixtures stored at 6 ± 2°C within 1 h prior to injection (p > 0.10). The mean LH IU for the LH standard was 70 IU/vial (range, 58.2–84.1). The results for the low and high dose mixtures were factored into the formula above.

**Table 2:** Luteinizing hormone (LH) bioactivities (mean and 95% confidence interval)

	Lutropin alfa (n = 36)	Lutropin alfa/follitropin alfa RFF mixture (1:1) (n = 30)	
LH bioactivity per vial (IU of NIBSC 98/704)	94.7 (78.7–113.8)	85.3 (70.8–102.6)	p > 0.10

RFF, revised formulation female.

## Discussion

For over 20 years, the mainstay of female infertility treatment was human menopausal gonadotropins (hMG), an injectable medication extracted from human urine with FSH and LH activity. During the 1980–90s, a host of technological improvements led to greater purity of urinary products and the availability of recombinant gonadotropins. The recombinant products differed from hMG in several significant ways: (1) purity, (2) mode of administration, e.g. self-administered by subcutaneous injection versus patient-assisted intramuscular injection, and (3) the presence of a single, well-characterized gonadotropin protein (r-hFSH, r-hLH, or r-hCG) [21]. The purity and characterization of recombinant gonadotropins paved the way for novel research on the roles of FSH and LH in follicular development and facilitated the independent titration of FSH and LH for the benefit of infertile patients [22].

Infertility treatment protocols are individualized according to a variety of factors, including the patient's age and diagnosis, ovarian reserve, history of prior response to COS, and co-administered medications. Due to the complexity of treatment, patients prefer the fewest number of steps to prepare each injection [23]. Accordingly, the need for administration of mixed doses of recombinant FSH and LH is becoming more apparent.

The results of the current study demonstrated that the biological potency of a mixture of 75 IU of follitropin alfa RFF (freeze-dried formulation) and 75 IU lutropin alfa in a 1:1 ratio, after reconstitution in SWFI using a plastic syringe, was similar to the biological potency of the individual respective recombinant hormones when administered within 1 h of injection and stored at 6 ± 2°C. As a result, this study has shown that follitropin alfa RFF and lutropin alfa may be mixed, without any significant alterations in either the resulting FSH or LH bioactivity, if administered within 1 h. It is important to note, however, that the results of this study cannot be extrapolated to other FSH or FSH-containing products, such as follitropin beta injection (a recombinant-derived product), urofollitropin for injection, purified or menotropins for injection, USP (urine-derived products). The urine-derived products are reconstituted with 2 ml 9% sodium chloride injection, USP. Follitropin beta injection is a 0.5 ml vial containing the active ingredient, excipients, and water for injection, USP.

The inherent lack of precision is a widely recognized shortcoming of the *in vivo *bioassays used to quantify gonadotropin content prior to release of commercial gonadotropin products [2,21]. The Steelman-Pohley *in vivo *bioassay used to determine FSH content is highly specific but it lacks precision, as the coefficient of variation (CV) in a single determination is 10–20% [24]. The USP, the official authority that establishes standards for the quality of all prescription products sold in the United States, describes the methods used to identify gonadotropin biopotency. The USP monograph specifies that these products contain ≥ 80% and ≤ 125% of the hormones listed on the products' labels [19]. As noted by Driebergen and Baer, realistically, a 75 IU vial of FSH could contain between 60 and 94 IU (80–125%) based on the bioassay, and between 48 and 117 IU based on the same bioassay's CV (10–20%) [24].

In the present study, the mean and 95% CI for follitropin alfa RFF alone was 84.2 IU (75.5–92.7), while that of the follitropin alfa RFF/lutropin alfa mixture was 87.6 IU (79.1–96.5). These results are consistent with the assay limitations previously discussed. Therefore, regardless of the product source (urine- or recombinant-derived), the FSH biopotency will vary because of the assay and not necessarily the FSH product itself. Because a highly consistent glycosylation profile is produced batch-to-batch, follitropin alfa RFF may be assessed through physico-chemical means [24].

The ovarian ascorbic acid depletion assay was used to assess the biopotency of LH in the current study. Although the Van Hell seminal vesicle weight gain bioassay is used by regulatory authorities to assess the LH content of gonadotropin products, the ovarian ascorbic acid depletion assay remains the most widely used *in vivo *bioassay for quantifying LH [25]. The ovarian ascorbic acid depletion assay is easy to perform and, furthermore, results correlate better than those of the seminal vesicle weight assay with *in vitro *LH assays [25,26]. However, the precision of the ovarian ascorbic acid depletion assay is limited by heterogeneity in the structure of LH, endogenous interferences, and inter-laboratory variability [25].

As previously described, le Cotonnec *et al*. evaluated the PK and PD interactions between 150 IU follitropin alfa and 150 IU lutropin alfa in a prospective, randomized crossover study in 12 healthy women [10]. No statistically significant differences were observed for t_max_, C_max_, or AUC between the single and combined doses for either r-hLH or r-hFSH. PD markers that measured the response to r-hFSH (serum estradiol and inhibin concentrations, and follicular number and size) were not markedly affected by r-hLH when both products were administered together. However, the formulations of follitropin alfa and lutropin alfa used by le Cotonnec *et al*. in this Phase I study differed from those currently marketed in the USA; the current formulations of both products include methionine and polysorbate 20 [12,17].

Reassuringly, the results of this study show that currently marketed formulations of follitropin alfa RFF and lutropin alfa may be mixed without significant alterations in the resulting bioactivity of either FSH or LH.

## Conclusion

Mixing follitropin alfa RFF and lutropin alfa in plastic syringes and administering the mixture within 1 h did not alter the bioactivity of either FSH or LH.

## Competing interests

Michael Alper is a consultant for EMD Serono, Inc., an affiliate of Merck KGaA, Darmstadt, Germany, and a member of the company's speakers' bureau. Diego Ezcurra, Joan Schertz and Eduardo Kelly are employees of EMD Serono, Inc., an affiliate of Merck KGaA, Darmstadt, Germany.

## Authors' contributions

RM, DE, MA, JS, and EK contributed to the study design. RM and CD conducted the research, and RM provided the statistical analysis. RM, DE, MA, and JS drafted the manuscript. All authors read and approved the final manuscript.
